# Four-factor prothrombin complex concentrate improves thrombin generation and prothrombin time in patients with bleeding complications related to rivaroxaban: a single-center pilot trial

**DOI:** 10.1186/s12959-017-0158-9

**Published:** 2018-01-10

**Authors:** Bettina Schenk, Stephanie Goerke, Ronny Beer, Raimund Helbok, Dietmar Fries, Mirjam Bachler

**Affiliations:** 1 Department of General and Surgical Intensive Care Medicine, Medical University of Innsbruck, Anichstr. 35, 6020 Innsbruck, Austria; 2 Department of Neurosurgery, Medical University of Innsbruck, Anichstr. 35, Innsbruck, 6020 Austria; 3 Department of Anatomy, Histology and Embryology, Division of Clinical and Functional Anatomy, Medical University of Innsbruck, Anichstr. 35, Innsbruck, 6020 Austria; 4 Department of Neurology, Medical University of Innsbruck, Anichstr. 35, 6020 Innsbruck, Austria; 5 Institute for Sports Medicine, Alpine Medicine and Health Tourism, UMIT - University for Health Sciences, Medical Informatics and Technology, Eduard Wallnöfer Zentrum 1, 6060 Hall in Tirol, Austria

## Abstract

**Background:**

Direct oral anticoagulants (DOACs) pose a great challenge for physicians in life-threatening bleeding events. The aim of this study was to test the efficacy of reversing the DOAC rivaroxaban using four-factor PCC (prothrombin complex concentrate), a non-specific reversing agent.

**Methods:**

Patients with life-threatening bleeding events during rivaroxaban treatment were included and administered 25 U kg^−1^ of PCC. Blood samples were collected immediately prior to as well as after PCC treatment at predefined time intervals. The primary endpoint was defined as the difference in thrombin generation (TG) parameters ETP (endogenous thrombin potential) and C_max_ (peak thrombin generation) prior to and ten minutes subsequent to PCC treatment.

**Results:**

Thirteen patients, of whom the majority suffered from intra-cranial haemorrhage (ICH) or subdural haemorrhage (SDH), were included and administered PCC. The results show that the ETP (TG) significantly (*p* = 0.001) improved by 68% and C_max_ (TG) by 54% (p = 0.001) during PCC treatment. In addition, the Quick value (prothrombin time: Quick^PT^) significantly improved by 28% and the activated partial thromboplastin time (aPTT) was decreased by 7% ten minutes after PCC administration. C_max_ was reduced at baseline, but not ETP, aPTT or Quick^PT^. Lag time until initiation (TG, t_lag_), thromboelastometry clotting time (CT_EXTEM_) and time to peak (TG, t_max_) correlated best with measured rivaroxaban levels and were out of normal ranges at baseline, but did not improve after PCC administration. In 77% of the patients bleeding (ICH/SDH-progression) ceased following PCC administration. During the study three participants passed away due to other complications not related to PCC treatment. The possibility of thrombosis formation was also evaluated seven days after administering PCC and no thromboses were found.

**Conclusions:**

This study shows that use of PCC improved ETP, C_max,_ Quick^PT^ and aPTT. However, of these parameters, only C_max_ was reduced at the defined baseline. It can be concluded that CT_EXTEM,_ t_lag_ and t_max_ correlated best with the measured rivaroxaban levels. The study drug was found to be safe. Nonetheless, additional studies specifically targeting assessment of clinical endpoints should be performed to further confirm these findings.

**Clinical trial registration:**

EudraCT trial No. 2013–004484-31.

**Electronic supplementary material:**

The online version of this article (10.1186/s12959-017-0158-9) contains supplementary material, which is available to authorized users.

## Background

Rivaroxaban (Xarelto®, Bayer, Germany) is used as thrombosis prophylaxis and therapy instead of anticoagulants such as unfractionated heparin, low molecular weight heparin or vitamin K antagonists, because it is considered to have a wider therapeutic range and a more predictable dose-response [1–3]. Although bleeding and bleeding complications in trauma and surgery are the most common complications/side-effects [4], no evidence-based reversal strategy is available for a bleeding patient taking rivaroxaban [5, 6]. A specific antidote called *Andexanet alfa* is in development, but not yet clinically available [7]. Therefore, the off-label use of coagulation factor concentrates, particularly four-factor prothrombin complex concentrate, in the management of rivaroxaban-related, life-threatening bleeding is the most recommended/used therapy [8, 9]: In healthy male volunteers, PCC was seen to reverse the anticoagulant effect of rivaroxaban [10, 11]. Especially for the treatment of intracranial haemorrhage (ICH), PCC seems to be a promising potential reversing agent [12–14]. However, managing life-threatening bleeding events under DOAC remains a challenge for physicians since in vivo data regarding efficacy of anticoagulation reversal are still lacking.

Therefore, the aim of this study was to evaluate the effect of PCC on coagulation status after life-threatening bleeding events in patients treated with rivaroxaban.

## Methods

### Ethics committee approval

This study was approved by the Human Subjects Review Board of the Medical University of Innsbruck, Austria (Ref.: UN2013–0048), as well as by the national competent authority (*Bundesamt für Sicherheit im Gesundheitswesen*, BASG, Vienna, Austria, Ref.: LCM-718199) and registered with EudraCT (Ref.: 2013–004484-31). Written informed consent was obtained from all study participants. Patients, who for medical reasons (unconscious, shock, sedated, etc.) were not able to give their consent to participate in this clinical trial, were enrolled based on the principle of deferred consent according to § 43a (1), Austrian Medicinal Products Law (“Österreichisches Arzneimittelgesetz”).

The study was performed in compliance with the Declaration of Helsinki guidelines regarding ethical principles for medical research involving human subjects and followed Good Clinical Practice as defined by the International Conference on Harmonization (ICH-GCP).

### Study design and setting

The present study is a single-centre, analytic, observational, prospective, open-labelled, single-armed, non-commercial clinical pilot trial.

### Selection of participants

Inclusion criteria were age over 18 years, patients receiving rivaroxaban and with life-threatening bleeding and the need for reversal and/or patients needing acute reversal of the anticoagulation effects of rivaroxaban. Patients who had already been administered pro-coagulant therapies including recombinant activated factor seven, activated prothrombin complex concentrates or other coagulation factor concentrates (except fibrinogen concentrate, DDAVP and tranexamic acid) were not included. Further exclusion criteria were: a greater risk for thromboembolic events than for bleeding, pregnancy, suspected or confirmed sepsis, recent history of thromboembolic events, and active participation in another clinical trial or refusal to participate.

### Interventions

The investigational medicinal product (four-factor PCC) was generously provided by CSL Behring, Marburg, Germany (Beriplex® P/N 500 IU). PCC was administered i.v. at a dosage of 25 IU/kg BW as bolus and/or continuous infusion. If bleeding continued after the first dosage, a second dosage of 25 IU/kg BW had to be administered. However, for all of the included patients only the first dosage of PCC was necessary. The maximum total allowed dose was 5000 IU.

Study assessments were performed as follows: Visit 1 (V1, baseline) was performed immediately before administration of four-factor PCC (25 IU/kg BW). Ten minutes after the end of PCC administration Visit 2 was conducted (V2). Thereafter, Visits were calculated as V3: +1 h, V4: +3 h, V5: +6 h, V6: +12 h, V7: +24 h, V8: +2 days, V9: +4 days, V10: +7 days, V11 + 30 days. At Visit 11 (30 days after V2) the patient was interviewed to evaluate his health status. Between days 5 and 7 an ultrasound examination of the leg arteries and veins was performed to screen for thrombosis. At every visit bleeding and thrombosis status were assessed, laboratory measurements performed and vital signs documented.

Blood coagulation assays were performed and blood coagulation factor concentrations determined using vacutainer tubes containing 1.106 mol l^−1^ trisodium citrate solution; blood samples for blood cell count were taken using vacutainer tubes containing 1.6 mg ml^−1^ 3 K EDTA. All blood sampling tubes were purchased from Sarstedt, Nümbrecht, Germany.

All coagulation analyses were performed immediately after blood draw, except thrombin generation. Plasma for thrombin generation assays was immediately frozen at −80 °C (or at −20 °C for a maximum of seven days) and thawed immediately before being analysed.

### Methods and measurements

ROTEM® parameters were determined using a ROTEM® gamma analyser (TEM Innovations GmbH, Munich, Germany). All tests were performed according to manufacturer’s instructions in whole blood immediately after blood sampling and using the specific liquid reagents provided by the manufacturer for EXTEM (extrinsically activated assay with tissue factor) and INTEM (intrinsically activated test using kaolin) measurements.

Thrombin generation measurements were performed using the Innovance ETP assay (Siemens, Marburg, Germany) on an automated coagulation analyser (BCS XP, Siemens, Marburg, Germany). Coagulation was activated by adding phospholipids, human recombinant tissue factor (in a concentration of 600–850 pmol l^−1^) and calcium ions to platelet-poor plasma. The generated thrombin cleaves a chromogenic substrate (H-b-Ala-Gly-Arg-pNA) and the turnover of the substrate is recorded over time. The final substrate concentration is 733 nmol l^−1^ and 19 mmol l^−1^ CaCl. The original curve is corrected for the estimated α macroglobulin-bound thrombin activity. From this curve the following parameters can be obtained: total amount of generated thrombin present in the reaction from the point of initiation until return to baseline, which is measured by the calculated increase in the extinction rate (measured in milli-extinctions or mE), and also known as “endogenous thrombin potential” (ETP), sometimes referred to as “area under the curve” (AUC), peak thrombin generation (C_max_) (the maximum of the first derivation of the ETP), lag phase until initiation (t_lag_), and time to peak thrombin activity (t_max_).

PT, aPTT, d-dimers, vWF, plasminogen, single factor levels (FII, FV, FVII, FVIII, FIX, FX, FXI, FXII and FXIII) and antithrombin activity were determined with proper reagents (PT: Thromborel® S; aPTT: Pathromtin® SL; D-Dimers: INNOVANCE® D-Dimer; vWF: vWF Ag®, Innovance® VWF Ac, Plasminogen: Berichrom® Plasminogen; factor II, V, VII, X: Factor-deficient plasma via Thromborel® S; factor VIII, IX, XI, XII: Factor-deficient plasma via Pathromtin® SL; antithrombin III Berichrom® Antithrombin III) on an automated coagulation analyser (reagents and BCSxp, Siemens, Marburg, Germany). The pondus Hydrogenii (pH) value was measured with proper reagents on an automated blood gas analyser (ABL800, Drott Medizintechnik GmbH, Austria). CRP was measured using an immunoturbidimetric assay (Tina-quant® CRPLX, Roche Diagnostics). Rivaroxaban levels were also measured on the BCSxp using a chromogenic assay calibrated for rivaroxaban (BIOPHEN® DiXa-I, CoaChrom Diagnostica, Neuville-sur-Oise, France). Reference ranges were taken from the respective product specification files of the test reagents used (compare above, all reagents and machines with CE certification) or from the respective standard references [15].

### Outcomes

Primary objective was assessment of the coagulation status after administration of PCC. Coagulation measurements included PT, aPTT, endogenous thrombin potential (ETP), thromboelastometry (ROTEM®), single factor concentrations, blood cell count and the rivaroxaban level.

Based on previous investigations [16–20], the primary endpoint was defined as the difference in thrombin generation parameters ETP and C_max_ between V1 and V2.

Secondary endpoints were the development of the following parameters from V1 to V10 and their correlation with measured rivaroxaban levels: thrombin generation, single factor profiles, standard coagulation tests PT, aPTT, fibrinogen, AT, and thromboelastometry (ROTEM®).

### Analysis

A descriptive analysis of all measured blood characteristics was performed. The Wilcoxon signed-rank test was used to evaluate the primary endpoint and all other differences in coagulation measurements between baseline and samples from the same patient at V2 to V10. To evaluate correlations between rivaroxaban level and blood coagulation assays and coagulation factor activities Pearson’s correlation was used. *P* values ≤0.05 were considered statistically significant. Statistical analyses were performed using STATISTICA 10 software (StatSoft Europe GmbH, Hamburg, Germany).

## Results

### Characteristics of study subjects

Fourteen patients (5 female, 9 male) aged between 47 and 96 years (median 80 years) were enrolled at the Medical University of Innsbruck. One patient (male) had to be excluded from analysis because he withdrew consent. Median weight was 76 kg (range 52 kg to 99 kg) and median height 170 cm (range 156 cm to 183 cm). Average body mass index was 25 (range 18 to 34).

The first visit to the first patient (FPFV) was in August 2014, last patient last visit (LPLV) was in October 2016. Underlying disease was mostly intra-cerebral haemorrhage (ICH, six patients), four patients suffered from subdural hematoma (SDH), one patient suffered from haemorrhage after removing the urinary catheter, one during laparotomy, and one suffered from gastrointestinal bleeding.

Prior medication (within seven days before V1), besides rivaroxaban (100%), was recorded if patients received additional antiplatelet medication (2 patients), procoagulatory medication (no patient) or anticoagulatory medication (no patient).

Median amount of administered PCC was 2000 IU (range 1500 to 2400 IU).

Concomitant medication (from V1 to V7), with potential influence on coagulation, included anticoagulant medication (12 patients), catecholamines (10 patients), antibiotics (6 patients), procoagulant medication (1 patient), anti-fibrinolytics (1 patient) and blood products (1 patient): ten patients received noradrenaline, seven patients enoxaparin, two patients rivaroxaban, and one patient each received danaparoid, fibrinogen concentrate, tranexamic acid and erythrocyte concentrate.

### Safety

Three subjects died during study participation, one from septic shock (multi-organ failure), one due to progressive cancer and one from ICH. All deaths were considered to be unrelated to the study medication. Of the thirteen study patients three showed signs of re-bleeding (progressive ICH) after administration of PCC. Other serious adverse events (SAEs) were an epileptic event, ischemia caused by thromboembolic closure of the femoral artery bifurcation (left), sepsis and embolic arteria cerebri anterior infarct (one patient). Both thromboembolic SAEs occurred only after day 7 (thrombotic screening via duplex ultrasound).

#### Main results

##### Baseline

For baseline values of laboratory parameters other than coagulation measurements, please refer to Table 1.

**Table 1:** Baseline laboratory parameters of all included patients

Parameter	Unit	Median	Min	Max	Reference range
Rivaroxaban	ng ml^−1^	103	20	425	
CRP^a^	mg dl^−1^	0.7	0.1	16.5	0.0–0.5
pH		7.42	7.33	7.49	7.37–7.45
FI (Clauss)	mg dl^−1^	303	240	524	210–400
FI (immun)	mg dl^−1^	365	272	591	200–400
ATIII (FIIa)	%	86	47	101	79–112
D-Dim^a^	μg l^−1^	994	191	3186	0–500
FII	%	115	65	146	70–120
FV	%	102	55	172	70–120
FVII	%	88	50	173	70–120
FVIII^a^	%	261	94	343	70–150
vWF^a^	%	288	149	509	58–174
FIX	%	104	65	161	70–120
FX	%	84	41	122	70–120
FXI	%	85	42	138	70–120
FXII	%	100	54	125	70–120
FXIII	%	116	49	130	70–120
Plasminogen	%	88	44	119	75–150
Leukocytes	×10^9^ l^−1^	8	3	13	4.0–10.0
Erythrocytes^a^	×10^12^ l^−1^	4.2	3.2	4.7	4.4–5.9
Haemoglobin	g l^−1^	131	93	141	130–177
Haematocrit^a^	l l^−1^	0.37	0.27	0.40	0.4–0.52
Platelets	×10^9^ l^−1^	172	84	344	150–380

*CRP* C-reactive protein, *pH* potential hydrogen, *FI* fibrinogen, blood coagulation factor I, *immun* immunological method, *AT-III* anti-thrombin III, *FI-FXIII* blood coagulation factors I-XIII, *D-Dim* d-dimers, *vWF* von Willebrand Factor. Highlighting: ^a^out of reference range

**Fig. 1:**
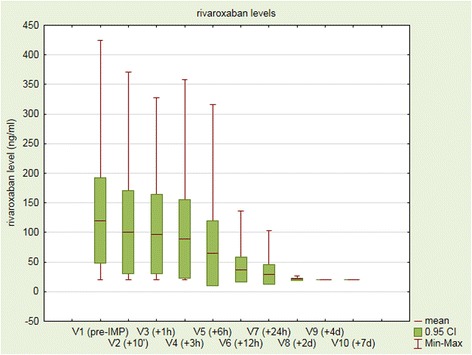
Means of rivaroxaban levels from V1 to V10 for all included patients. Lowest rivaroxaban levels are indicated as 20 ng ml^−1^, because a lower level is not detectable

Mean rivaroxaban level at V1 was 120 ng ml^−1^ (95% confidential interval 48 to 192 ng ml^−1^). The course of measured rivaroxaban levels can be seen in Fig. 1. Standard laboratory parameters aPTT and PT (Quick value) were in normal range. Thromboelastometry EXTEM clotting time (CT_EXTEM_) was prolonged, C_max_ (TG) was reduced and ETP (TG) was in normal range. Moreover, C-reactive protein (CRP) levels were increased, as were d-dimers, FVIII levels and vWF levels, whereas erythrocyte and leukocyte counts were below reference ranges for healthy subjects. Blood loss was 800 and 750 cm^3^ for patients who were bleeding externally, whereas in the case of ICH intracerebral blood volume was 0.02 cm^3^, 8 cm^3^, 12 cm^3^, 34 cm^3^, 43 cm^3^ and 99 cm^3^, respectively. Regarding SDH, blood loss volume was 3 cm^3^, 11 cm^3^, 33 cm^3^ and 116 cm^3^, respectively. In the case of gastrointestinal bleeding blood loss was not measured. All baseline coagulation laboratory measurements can be seen from Table 2.

**Table 2:** Coagulation parameters from V1 to V10

Parameter	aPTT	Quick^PT^	ETP	C_max_	t_lag_	t_max_	CT_EXTEM_	CT_INTEM_
Unit	sec	%	mE	mE min^−1^	sec	sec	sec	sec
Ref. range	26–37	70–130	312–441	111–156	19.6–25.6	50.8–72.0	42–78	134–218
V1	30 (6)	72 (16)	316 (75)	80 (31)^*^	52 (70)^*^	99 (67)^*^	94 (31)^*^	178 (37)
V2	28 (7)^‡^	92 (14)^‡^	524 (94)^‡*^	123 (25)^‡^	41 (12)^*^	95 (24)^*^	96 (34)^*^	198 (48)
V3	29 (6)^‡^	89 (11)^‡^	502 (104)^‡*^	114 (28)^‡^	39 (9)^*^	99 (29)^*^	91 (25)^*^	180 (41)
V4	33 (5)	87 (13)^‡^	518 (92)^‡*^	112 (26)^‡^	37 (7)^*^	93 (25)^*^	95 (25)^*^	193 (29)
V5	32 (6)	88 (15)^‡^	544 (102)^‡*^	127 (22)^‡^	33 (6)^*^	91 (25)^*^	83 (22)^*^	170 (37)
V6	32 (11)	83 (18)^‡^	528 (118)^‡*^	131 (31)^‡^	34 (9)^*^	83 (29)^*^	80 (17)^*^	176 (27)
V7	33 (6)	79 (13)^‡^	466 (72)^‡*^	115 (24)	31 (5)^*^	82 (16)^*^	82 (17)^*^	185 (32)
V8	31 (6)	88 (15)^‡^	411 (98)^‡^	111 (27)	27 (5)^*^	69 (16)	62 (15)	156 (37)
V9	34 (6)	96 (16)^‡^	380 (96)	95 (26) ^‡*^	28 (3)^*^	64 (4)	53 (20)	163 (28)
V10	29 (7)	88 (22)	371 (98)	103 (26)^*^	26 (4)^*^	59 (5)	70 (17)	156 (37)

*aPTT* activated partial thromboplastin time, *Quick*^*PT*^ Quick value, prothrombin time, *mE* milli extinctions, *ETP* endogenous thrombin potential, *C*_*max*_ thrombin generation, peak thrombin generation; *t*_*lag*_ thrombin generation, lag time until initiation, *t*_*max*_ thrombin generation, time to peak thrombin activity, *CT*, ROTEM®, clotting time, *EXTEM* extrinsic coagulation pathway; INTEM, intrinsic clotting time. Wilcoxon signed-rank test was used to evaluate differences between baseline and samples from the same patient at V2-V10 (indicated as ^‡^). Values are indicated as mean values (+/− standard deviation). Highlighting: ^*^out of reference range, ^‡^significantly changed after PCC administration (as compared to V1)

### Primary endpoint

**Fig. 2:**
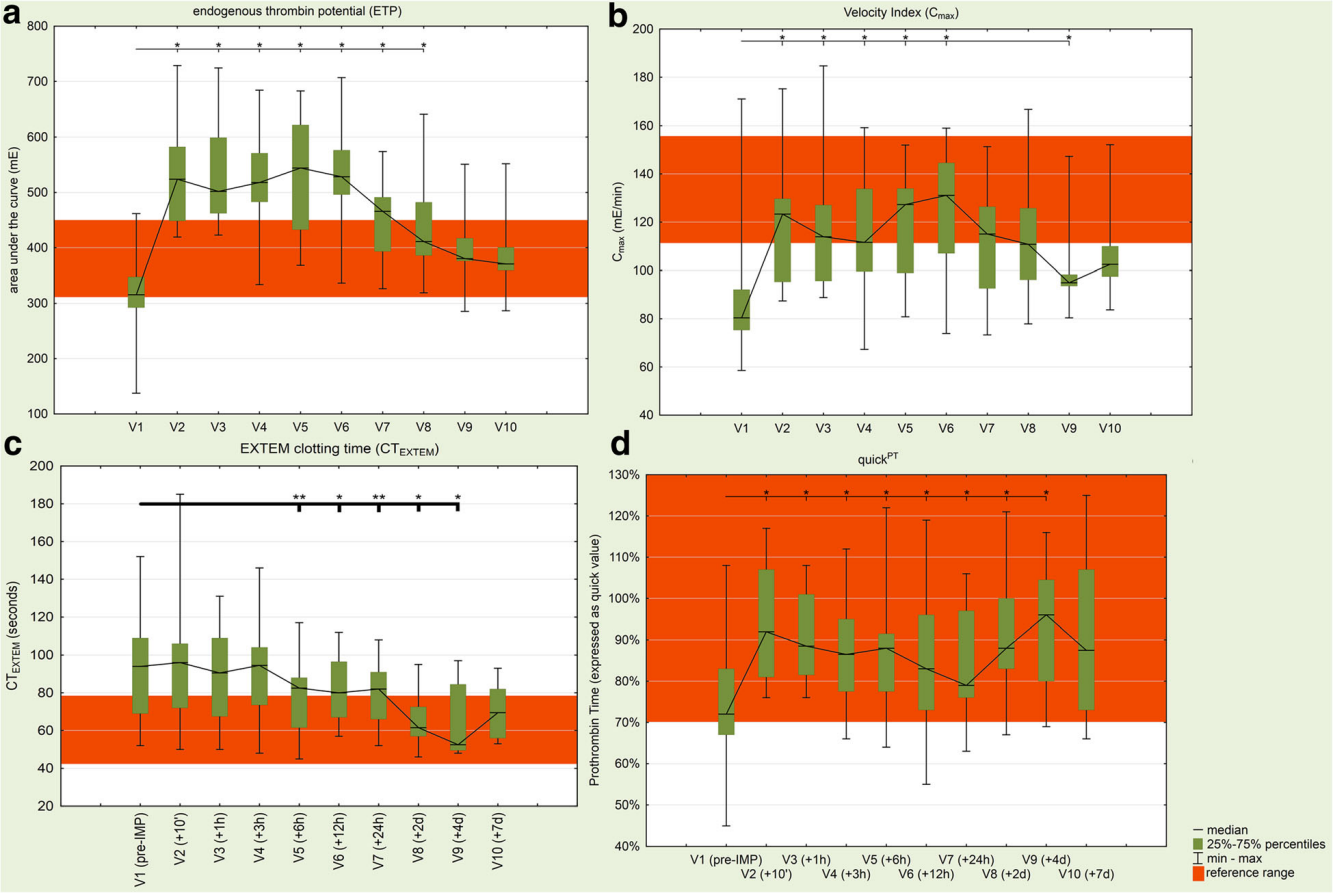
Effect of four-factor PCC on coagulation parameters. ETP (**a**), C_max_ (**b**), CT_EXTEM_ (**c**) and Quick^PT^ (**d**) for all patients from V1 to V10. Normal (healthy persons without anticoagulation) values are indicated (red area). Significant differences from baseline were determined using the Wilcoxon signed-rank test (*p < 0.05, **p < 0.01). Abbreviations: ETP, endogenous thrombin potential; C_max_, thrombin generation, peak thrombin generation; CT, ROTEM®, clotting time; EXTEM, ROTEM®, extrinsic coagulation pathway; Quick^PT^, Quick value (prothrombin time)

All included patients reached the primary endpoint (difference in thrombin generation between V1 and V2). The ETP significantly (*p* = 0.001) increased by 68% as a result of administration of PCC (median amount of administered PCC: 2000 IU [range 1500–2400 IU]) from 316 mE at V1 to 530 mE at V2. C_max_ significantly (*p* = 0.001) improved by 54% from 80 mE min^−1^ (V1) to 123 mE min^−1^ (V2). t_lag_ and t_max_ did not show a significant difference between V1 and V2 (Fig. 2a and b).

### Secondary endpoints

Regarding thrombin generation in addition to reaching the primary endpoint (compare above), the ETP remained significantly elevated until 48 h after PCC administration (V8) and C_max_ until 12 h after PCC administration (V6). Compare Table 1 and Fig. 2a/b.

None of the measured ROTEM® parameters significantly improved after administration of PCC. MCF_INTEM_ significantly decreased by 3% (*p* = 0.019) after PCC administration and remained significantly decreased until one hour after PCC administration (V3, data not shown). CT_EXTEM_ was significantly decreased only after V5 (compare Fig. 2c). Assessment of coagulation status following the administration of PCC showed a significant increase in Quick^PT^ (+28%) ten minutes after PCC administration (V2). The Quick value remained significantly increased, as compared to baseline (V1), until V9 or four days after administration of PCC (compare Fig. 2d). Moreover, PCC caused a significant decrease of 7% in aPTT. aPTT also remained significantly decreased until V2 (compare Table 2).

The single factor profiles showed a significant increase in activation of all coagulation factors contained in PCC (factors II, VII, IX and X) until V3 (FVII), V4 (FII) and until V7 (FIX and FX) as compared to baseline V1. All other coagulation factors showed no increase in activity following PCC administration (compare Additional file 1**:** Table S1).

Regarding correlation of rivaroxaban level with coagulation parameter, best correlation was found between t_lag_ and rivaroxaban level (single measurements for every patient from V1 to V10 correlated with the respective rivaroxaban level) followed by CT_EXTEM_, CT_INTEM_, Quick value, and C_max_, MCF_EXTEM_, and t_lag_. All other coagulation parameters showed no significant correlation with rivaroxaban levels (compare Table 3).

**Table 3:** Correlations between rivaroxaban concentrations and blood coagulation parameters

Individual values			Mean values		
parameter	r	p	parameter	r	p
t_max_	0.465	<0.001	t_lag_	0.924	<0.001
CT_EXTEM_	0.355	<0.001	CT_EXTEM_	0.916	<0.001
CT_INTEM_	0.296	0.002	t_max_	0.902	<0.001
Quick^PT^	−0.294	0.002	MaxV-t_FIBTEM_	0.887	0.001
C_max_	−0.242	0.019	MaxV-t_EXTEM_	0.819	0.004
MCF_EXTEM_	0.238	0.015	MCF_FIBTEM_	0.779	0.008
t_lag_	0.222	0.027	CT_INTEM_	0.742	0.014
MaxV-t_FIBTEM_	0.221	0.128	FI_clauss_	0.701	0.024
MCF_INTEM_	0.203	0.037	MaxV-t_INTEM_	0.598	0.068
CFT_EXTEM_	0.194	0.047	FI_immun_	0.579	0.080
MaxV-t_EXTEM_	0.172	0.242	ATIII_Xa_	0.533	0.112
CFT_INTEM_	0.139	0.156	MCF_INTEM_	0.423	0.224
aPTT	0.108	0.267	Quick^PT^	0.340	0.337
MaxV-t_INTEM_	0.070	0.635	CFT_INTEM_	0.328	0.355
ETP	0.025	0.809	CFT_EXTEM_	0.266	0.457
MCF_FIBTEM_	0.005	0.963	aPTT	0.185	0.609

Individual values (left side, individual values for every single patient and for the respective test that was measured at every time point from V1 to V10) or mean values (right side, mean values of all patients and mean values of the measured rivaroxaban levels from V1 to V10) of all coagulation tests for all patients at every visit were correlated with the respective rivaroxaban concentrations at the same visit. The Pearson correlation was used to detect correlations between various blood coagulation tests and rivaroxaban concentrations. Tests were sorted according to the strength of correlation (r). Abbreviations: *t*_*max*_ thrombin generation, time to peak, *CT, ROTEM®* clotting time, *EXTEM*, ROTEM® extrinsic coagulation pathway, *INTEM* ROTEM®, intrinsic clotting time, *Quick*^*PT*^ Quick value (prothrombin time), *C*_*max*_ thrombin generation, peak thrombin generation, *MCF* ROTEM®, maximum clot firmness; *t*_*lag*_ thrombin generation, lag time until initiation, *MaxV − t* ROTEM®, time from reaction start until the maximum of the first derivate of the curve is reached, *FIBTEM* ROTEM®, fibrinogen-dependent coagulation, *CFT* ROTEM®, clot formation time, *aPTT* activated partial thromboplastin time, *ETP* endogenous thrombin potential

**Fig. 3:**
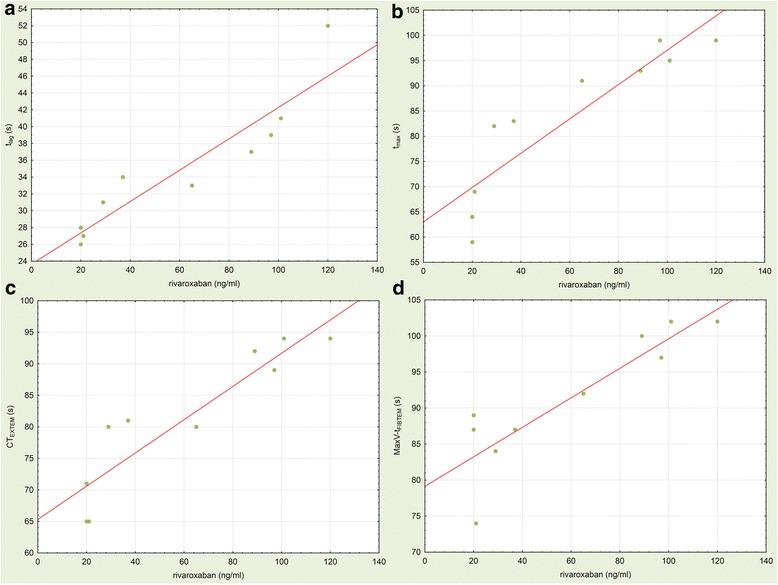
Correlations between means of rivaroxaban plasma levels and various coagulation parameters from V1 to V10. Significantly (*p* < 0.05 for all) correlated parameters were (**a**) thrombin generation lag time (t_lag_, linear approximation for t_lag_ = 23.63 + 0.19*x, *r* = 0.924, *p* < 0.001); (**b**) thrombin generation time to peak (t_max_, linear approximation for t_max_ = 63.02 + 0.34*x, *r* = 0.903, *p* < 0.001); (**c**) EXTEM clotting time (CT_EXTEM_, linear approximation for CT = 65.312 + 0.26*x, *r* = 0.926, p < 0.001) and (**d**) thromboelastometry FIBTEM time to maximum velocity (MaxV-t_FIBTEM_, linear approximation for MaxV-t_FIBTEM_ = 79.132 + 0.205*x, *r* = 0.887, *p* < 0.001). Strength of correlation was interpreted by evaluating the correlation coefficient: r 0.9–1.0, very strong correlation; r 0.7–0.89, strong correlation; r 0.5–0.69, moderate correlation; r 0.3–0.49, weak correlation

When comparing mean values of rivaroxaban and the mean values of all measured coagulation parameters at every time point (V1 – V10), t_lag_, CT_EXTEM_, t_max_, MaxV-t_FIBTEM_, MaxV-t_EXTEM_, MCF_FIBTEM_, CT_INTEM_ and FI_clauss_ showed a significant correlation with rivaroxaban levels. All other coagulation parameters showed no significant correlations with rivaroxaban levels (compare Table 3**,** Fig. 3). For values of all measured laboratory parameters, please refer to Additional file 1**:** Table S1.

## Discussion

The present study enrolled patients with the need for acute reversal of the effects of rivaroxaban to investigate the effects of PCC on the coagulation status. The majority of included patients (77%) suffered from life-threatening bleeding events in the central nervous system (ICH or SDH). Administration of PCC resulted in improved thrombin generation (C_max_, ETP), aPTT and Quick^PT^, whereas ETP, aPTT and Quick^PT^ were in normal range already before treatment. Therefore, C_max_ was the only parameter reduced at baseline (out of normal range) that improved by PCC administration. T_lag_, CT_EXTEM_ and t_max_ correlated best with measured rivaroxaban levels and were out of normal ranges at baseline, but did not respond to PCC administration. Study design and the small number of included patients do not allow a profound safety evaluation to be conducted. However, ultrasound examination seven days after administration of four-factor PCC revealed no thrombotic events. The occurrence of thrombosis found in the present study was 14% during the observation period (30 days), which is comparable to the frequency found in the ANNEXA-4 study with an incidence of 18% [21].

To date, no studies regarding the efficacy of PCC in patients with life-threatening bleeding events, associated with DOACs, are available. Yoshimura and colleagues recently published data from an observational study evaluating PCC in ten patients with bleeding complications associated with DOAC treatment: PT also significantly improved after PCC administration, but in contrast to our study they found no significant changes in aPTT [22]. This might be attributed to the lower dose of PCC administered (median 1000 IU vs. 2000 IU in the present study). A recent retrospective study by Grandhi and colleagues investigated the effect of PCC on ICH in 16 patients under rivaroxaban treatment. They collected no data about coagulation status but found PCC to be effective in reducing haemorrhagic complications and hematoma expansion [12]. Erenberg and colleagues conducted a randomized, double-blind, placebo-controlled study with 12 healthy volunteers who received rivaroxaban for two and a half days (2 × 20 mg) and subsequently a PCC bolus (50 U kg^−1^): effects of rivaroxaban and PCC were evaluated using the PT and ETP. Both parameters were affected by rivaroxaban and improved by PCC [10]. A similar study set up by Levi and colleagues with 35 healthy volunteers, who were randomized into a 4-factor PCC, a 3-factor PCC (50 U kg^−1^ for both) and a placebo (saline) group after four days of rivaroxaban (2 × 20 mg) treatment, also revealed that 4-factor PCC improved PT, aPTT and ETP [18]. In a recent study, we already demonstrated the effects of PCC on CT_EXTEM_ and TG ex vivo [16]: PCC was able to improve both parameters ex vivo, and rivaroxaban levels correlated with Quick^PT^, CTs, C_max_, aPTT and t_lag_.

The main difference between the present study and previous investigations is the different study set-up (in vivo). Consequently, rivaroxaban levels were more heterogeneous than in controlled ex vivo studies with patient blood or in vitro studies with healthy volunteers. For example, in the present study ETP was not reduced (as compared to normal values) at baseline V1, but the exact time of rivaroxaban intake was mostly not known. Consequently, rivaroxaban levels were lower than in comparable studies and ETP was not impaired.

The majority of patients were admitted with acute intracerebral haemorrhage. Early hematoma enlargement occurs more frequently in patients on anticoagulant treatment, and is associated with worsened long-term outcome [23]. Therefore, immediate reversal of any anticoagulant is recommended. Although we did not include a clinical or radiographic outcome parameter in the current study, we were able to show that PCC can be used to improve coagulation in these patients.

Interestingly, factor VIII and VWF levels were shown to be strongly increased (>200%) at all investigated time points. We already observed this phenomenon in previous investigations with patients who were not bleeding under rivaroxaban [16]. A potential explanation is that blood coagulation factor VIII and VWF levels significantly increase during an acute phase response [24].

Limitations of the present study are the lack of randomization / a control group and the small number of patients, resulting in insufficient data on mortality or effective haemostasis. However, this is the first prospective clinical study investigating the effect of PCC on rivaroxaban-related, life-threatening bleeding in vivo. Previous investigations have focused on healthy volunteers receiving an oral Xa inhibitor followed by PCC. To obtain these data at precise time points in emergency patients is challenging and not possible in a large number of patients except in an industry-sponsored study with research staff available around the clock. Especially the measured laboratory parameters add valuable knowledge to this topic.

Nevertheless, great care has to be taken since patients concomitantly took other medication, which probably influenced the results. Moreover, the influence of rivaroxaban on PT, aPTT, coagulation factor levels and thromboelastometry assays differs widely depending on the reagents used and must be evaluated for each particular reagent. Consequently, effects of rivaroxaban on the various assays can also be expected to differ from reagent to reagent.

Consequently, additional studies focusing on these outcomes must be conducted. Even though a specific antidote for the reversal of rivaroxaban is in development, there is still little knowledge about the real efficacy of Andexanet Alpha: preliminary analysis of the ANNEXA-4 study showed 79% efficacy. However, four hours after administration of the antidote rivaroxaban levels returned almost to pre-treatment values [21]. Moreover, it is still unclear whether smaller hospitals will be able to store these, presumably very expensive, antidotes. Additionally, specific antidotes that directly target and eliminate the anticoagulant activity of the DOACs do not possess pro-coagulant properties, which might be necessary in patients suffering from severe haemorrhage. This indicates that coagulation factor concentrates should still be part of the management strategy in bleeding patients.

## Conclusions

No controlled clinical studies in humans involving potential reversing agents in bleeding situations are available yet, which illustrates a difficulty in evaluating the potential reversing agents and proposing them for the management of life-threatening bleeding in rivaroxaban-anticoagulated patients. Our data indicate that four-factor PCC might be a valuable alternative to specific antidotes for the reversal of rivaroxaban-induced and life-threatening bleeding events. Further studies with clinical endpoints will have to confirm these findings.

## Additional file

Additional file 1: Table S1. Data are indicated as means (+/−standard deviation). Abbreviations: CRP, C-reactive protein; FI, fibrinogen; immun, immunologic method; ATIII, anti-thrombin III; FIIa, activated blood coagulation factor IIa; FXa, activated blood coagulation factor Xa; FII, blood coagulation factor II; FV, blood coagulation factor V, etc.; vWF, Von Willebrand factor. (DOCX 23 kbr)
