# Supplementary material for: Four-factor prothrombin complex concentrate improves thrombin generation and prothrombin time in patients with bleeding complications related to rivaroxaban: a single-center pilot trial
Source: Thromb J. 2018 Jan 10;16:1. doi: 10.1186/s12959-017-0158-9 (PMC5763793; doi:10.1186/s12959-017-0158-9)
Supplement: Additional file 1: Table S1. — Data are indicated as means (+/−standard deviation). Abbreviations: CRP, C-reactive protein; FI, fibrinogen; immun, immunologic method; ATIII, anti-thrombin III; FIIa, activated blood coagulation factor IIa; FXa, activated blood coagulation factor Xa; FII, blood coagulation factor II; FV, blood coagulation factor V, etc.; vWF, Von Willebrand factor. (DOCX 23 kbr) [file 12959_2017_158_MOESM1_ESM.docx]

**Supplementary Table 1 *All measured laboratory parameters from V1 to V10* *of all included patients.***

| **Parameter** | **Unit** | **V1** | **V2** | **V3** | **V4** | **V5** | **V6** | **V7** | **V8** | **V9** | **V10** |
| --- | --- | --- | --- | --- | --- | --- | --- | --- | --- | --- | --- |
| **CRP** | mg dl^-1^ | 0.69 (5.33) | 0.95 (5.56) | 0.85 (5.48) | 0.85 (6.24) | 0.95 (6.03) | 1.4 (8.19) | 1.2 (11.17) | 3.55 (8.04) | 2.7 (6.99) | 1.29 (3.86) |
| **FI_Clauss_** | mg dl^-1^ | 303 (80) | 300 (86) | 311 (88) | 280 (100) | 311 (97) | 317 (79) | 348 (84) | 343 (79) | 431 (84) | 388 (100) |
| **FI_immun_** | mg dl^-1^ | 365 (106) | 344 (105) | 335 (126) | 316 (120) | 362 (112) | 336 (128) | 348 (115) | 386 (87) | 489 (114) | 429 (113) |
| **ATIII^FIIa^** | % | 86 (17) | 79 (16) | 75 (18) | 73 (19) | 72 (19) | 69 (19) | 69 (19) | 71 (13) | 74 (19) | 72 (21) |
| **rivaroxaban** | ng ml^-1^ | 103 (119) | 69 (110) | 51 (105) | 53 (105) | 31 (86) | 22 (33) | <20 (25)* | <20 (2)* | <20 (0)* | <20 (0)* |
| **d-dimers** | µg l^-1^ | 994 (1053) | 968 (1233) | 1,291 (3331) | 1,105 (1823) | 1,440 (3398) | 1,498 (4030) | 1,852 (4686) | 2,617 (1408) | 2,058 (6842) | 3,062 (3258) |
| **FII** | % | 115 (26) | 144 (29) | 137 (20) | 132 (27) | 124 (35) | 119 (33) | 119 (37) | 117 (34) | 111 (31) | 106 (23) |
| **FV** | % | 102 (27) | 105 (29) | 112 (23) | 102 (23) | 100 (27) | 98 (40) | 101 (33) | 130 (37) | 134 (52) | 131 (35) |
| **FVII** | % | 88 (33) | 108 (26) | 109 (20) | 99 (30) | 89 (28) | 81 (29) | 77 (26) | 95 (26) | 79 (38) | 90 (32) |
| **FVIII** | % | 261 (74) | 255 (75) | 255 (73) | 226 (72) | 228 (71) | 201 (86) | 247 (86) | 225 (61) | 260 (114) | 309 (76) |
| **vWF** | % | 288 (107) | 286 (103) | 291 (88) | 242 (105) | 232 (107) | 261 (100) | 238 (125) | 288 (74) | 291 (122) | 403 (103) |
| **FIX** | % | 104 (24) | 128 (26) | 124 (20) | 123 (20) | 118 (33) | 114 (28) | 117 (31) | 130 (21) | 118 (35) | 145 (28) |
| **FX** | % | 84 (26) | 144 (25) | 128 (24) | 122 (24) | 116 (32) | 104 (32) | 92 (31) | 98 (29) | 73 (30) | 98 (24) |
| **FXI** | % | 85 (27) | 94 (25) | 90 (22) | 76 (23) | 83 (24) | 76 (29) | 80 (39) | 84 (25) | 89 (26) | 106 (23) |
| **FXII** | % | 100 (23) | 103 (21) | 91 (22) | 89 (23) | 83 (26) | 90 (29) | 95 (33) | 94 (26) | 74 (29) | 91 (36) |
| **FXIII** | % | 116 (27) | 103 (24) | 91 (26) | 93 (28) | 98 (29) | 94 (28) | 75 (34) | 87 (30) | 78 (45) | 77 (39) |
| **plasminogen** | % | 88 (18) | 87 (21) | 86 (19) | 88 (23) | 82 (24) | 75 (23) | 85 (24) | 80 (19) | 94 (24) | 89 (22) |
| **leukocytes** | G l^-1^ | 8 (3) | 9 (3) | 9 (3) | 8 (2) | 7 (3) | 8 (3) | 9 (4) | 8 (3) | 8 (4) | 8 (5) |
| **erythrocytes** | T l^-1^ | 4 (0.5) | 4 (0.4) | 4 (0.4) | 4 (0.5) | 4 (0.7) | 4 (0.4) | 4 (0.5) | 3 (0.6) | 4 (0.6) | 4 (0.5) |
| **haemoglobin** | G l^-1^ | 131 (16) | 128 (13) | 124 (13) | 115 (16) | 114 (23) | 116 (14) | 115 (17) | 109 (19) | 121 (17) | 117 (16) |
| **haematocrit** | l l^-1^ | 0.37 (0.04) | 0.37 (0.03) | 0.37 (0.03) | 0.37 (0.04) | 0.37 (0.06) | 0.37 (0.04) | 0.37 (0.05) | 0.37 (0.05) | 0.37 (0.05) | 0.37 (0.04) |
| **platelets** | G l^-1^ | 172 (82) | 163 (89) | 165 (89) | 174 (81) | 160 (82) | 177 (77) | 180 (90) | 194 (69) | 157 (68) | 167 (73) |

***Supplementary Table 1 legend:*** *Data are indicated as means (+/-standard deviation). Abbreviations: CRP, C-reactive protein; FI, fibrinogen; immun, immunologic method; ATIII, anti-thrombin III; FIIa, activated blood coagulation factor IIa; FXa, activated blood coagulation factor Xa; FII, blood coagulation factor II; FV, blood coagulation factor V, etc.; vWF, Von Willebrand factor.*
